# Continuous block at the proximal end of the adductor canal provides better analgesia compared to that at the middle of the canal after total knee arthroplasty: a randomized, double-blind, controlled trial

**DOI:** 10.1186/s12871-020-01165-w

**Published:** 2020-10-09

**Authors:** Yuda Fei, Xulei Cui, Shaohui Chen, Huiming Peng, Bin Feng, Wenwei Qian, Jin Lin, Xisheng Weng, Yuguang Huang

**Affiliations:** 1 Anesthesiology Department, Peking Union Medical College Hospital, Chinese Academy of Medical Sciences, and Peking Union Medical College, Shuaifuyuan 1#, Dongcheng District, Beijing, 100730 China; 2 Orthopaedic Department, Peking Union Medical College Hospital, Chinese Academy of Medical Sciences, and Peking Union Medical College, Shuaifuyuan 1#, Dongcheng District, Beijing, 100730 China

**Keywords:** Opioid-sparing, Total knee anthroplasty, Adductor canal block, Analgesia, Sufentanil

## Abstract

**Background:**

The optimal position for continuous adductor canal block (ACB) for analgesia after total knee anthroplasty (TKA) remians controversial, mainly due to high variability in the localization of the the adductor canal (AC). Latest neuroanatomy studies show that the nerve to vastus medialis plays an important role in innervating the anteromedial aspect of the knee and dives outside of the exact AC at the proximal end of the AC. Therefore, we hypothesized that continuous ACB at the proximal end of the exact AC could provide a better analgesic effect after TKA compared with that at the middle of the AC (which appeared to only block the saphenous nerve).

**Methods:**

Sixty-two adult patients who were scheduled for a unilateral TKA were randomized to receive continuous ACB at the proximal end or middle of the AC. All patients received patient-controlled intravenous analgesia with sufentanil postoperatively. The primary outcome measure was cumulative sufentanil consumption within 24 h after the surgery, which was analyzed using Mann-Whitney U tests. *P*-values < 0.05 (two-sided) were considered statistically significant. The secondary outcomes included postoperative sufentanil consumption at other time points, pain at rest and during passive knee flexion, quadriceps motor strength, and other recovery related paramaters.

**Results:**

Sixty patients eventually completed the study (30/group). The 24-h sufentanil consumption was 0.22 μg/kg (interquartile range [IQR]: 0.15–0.40 μg/kg) and 0.39 μg/kg (IQR: 0.23–0.52 μg/kg) in the proximal end and middle groups (*P* = 0.026), respectively. There were no significant inter-group differences in sufentanil consumption at other time points, pain at rest and during passive knee flexion, quadriceps motor strength, and other recovery related paramaters.

**Conclusions:**

Continuous ACB at the proximal end of the AC has a better opioid-sparing effect without a significant influence on quadriceps motor strength compared to that at the middle of the AC after TKA. These findings indicates that a true ACB may not produce the effective analgesia, instead, the proximal end AC might be a more suitable block to alleviate pain after TKA.

**Trial registration:**

This study was registered at ClinicalTrials.gov (NCT03942133; registration date: May 06, 2019; enrollment date: May 11, 2019).

## Background

Severe pain is common after total knee anthroplasty (TKA), especially in the first 24 h postoperatively and during active range of motion [1], which may span from 2 ~ 3 days and significantly limit early mobilization, rehabilitation, and recovery [2, 3]. Continuous adductor canal block (ACB) is recommended as an analgesic method for early postoperative pain treatment after TKA as it preserves quadriceps strength compared with continuous femoral nerve block. Continuous ACB also provides better analgesia compared with single ACB [4].

The optimal location for continuous ACB for TKA has been investigated by previous randomized clinical trials (RCTs) [5–8]. However, identification of the adductor canal (AC) was not consistent [5–8], and the results differed. The AC is a musculoaponeurotic tunnel that runs proximally from the apex of the femoral triangle (FT)/proximal end (entrance opening) of the AC where the medial borders of the sartorius muscle (SM) and adductor longors muscle (ALM) align, to the adductor hiatus distally where the femoral artery (FA) diverges from the SM and becomes deep [9]. The internal landmarks defined above can be easily identified via ultrasound, which has recently been deemed to be a more accurate and reliable method to identify the exact location of the AC [10–12]. However, to the best of our knowledge, the ideal continuous ACB location (for analgesia after TKA) of the true AC identified with these sonographic landmarks has not been investigated in a clinical setting.

Inside the AC, the neurovascular bundle is situated between the adductor muscles (longus and magnus) posteromedially, the medial vastus muscle anterolaterally, and the vastoadductor membrane anteromedially [10–12]. Studies which have investigated the relevant neuroanatomy of the thigh and knee found that the saphenous nerve (SN) that innervates the anteriomedia of the knee is the only nerve that is consistently found in the AC [10, 13, 14]. The nerve to vastus medialis (NVM), a femoral nerve branch which also plays an important role in the inervation of the anteromedial aspect of the knee [10, 15–17], though described in anatomical textbooks as being within the AC, has been recently shown to dive into a fascial tunnel, proximal to the entrance of the AC, between the medial vastus muscle and the ALM outside the AC in 90% of humans [13, 18, 19]. Indeed, previous cadaveric studies by Andersen et al. and, more recently, by Johnston et al. found that injectates administered into the AC or the distal AC could only capture the SN [18, 20]. In contrast, when the injectates were administered into the distal FT, both the SN and NVM were stained [19, 20]. Other investigators speculated that “a true ACB may not produce effective analgesia after TKA if the NVM is an important contributor to knee innervation” [12].

We therefore conducted this clinical trial to test the hypothesis that during continuous ACB, postoperative analgesia after TKA would improve with the catheter tip inserted at the less studied proximal end of the true AC, compared with a more distal locaion at the middle of the AC. The primary outcome was the median sufentanil consumption 24 h after surgery.

## Methods

### Enrollment

This study was approved by the Institutional Review Board of Peking Union Medical College Hospital in Beijing, China (#ZS-1030) and was registered at ClinicalTrials.gov (NCT03942133; date of registration: May 06, 2019; date of patient enrollment: May 11, 2019). Written informed consent was obtained from all participants before taking part. This manuscript adheres to the applicable Consolidated Standards of Reporting Trials guidelines and was conducted in accordance with the Declaration of Helsinki. Adult (≥18 years of age) patients with an American Society of Anesthesiologists (ASA) physical status classification of I to III who were scheduled for unilateral, primary TKA were approached for inclusion. Exclusion criteria were a body mass index (BMI) > 40, contraindications to peripheral nerve blocks, known daily intake of opioids (morphine, oxycodone, methadone, ketobemidone, fentanyl), alcohol or drug abuse, intolerance of nonsteroidal anti-inflammatory drugs, diabetes, lower limb neuropathy, and the inability to accurately describe postoperative pain to the investigators (e.g., a language barrier or a neuropsychiatric disorder).

### Randomization and blinding

Participants were randomized to either the proximal end or middle group with a ratio of 1:1 using a computer-generated sequence given by a professional statistician who was not otherwise involved in the study. Allocation concealment was ensured by the use of sealed, opaque, sequentially numbered envelopes which remained concealed until the block was performed.

All the ultrasound-guided continuous ACBs were conducted by a single senior experienced staff anesthesiologist (C.X.) in a dedicated procedure room, where all other surgeons, nurses (except the assistant research nurse in the procedure room), and study participants were not presented at the time of performing the block. Surgeries were conducted by the same surgical team blinded to subject allocation using a standardized approach.

### Perioperative management

All recruited subjects were interviewed on the day before surgery. Baseline pain severity and quadriceps strength of the operative leg were recorded. Subjects were informed of the postoperative continuous ACB and patient-controlled intravenous analgesia (PCIA) schedule, with a goal of maintaining pain scores < 4 on an 11-point numerical rating scale (NRS, 0: no pain; 10: maximum pain imaginable). No preoperative medications were administered.

### Catheter insertion procedure

All perineural catheter insertions were performed 40 min before surgery in a dedicated procedure room. Standard monitoring and peripheral venous access were established. Patients were placed in a supine position with the operative knee slightly flexed and externally rotated. With the ultrasound screen facing away from the patient, an ultrasound scan was carried out with a 13–6 MHz linear probe (Sonosite X-port, SonoSite Inc., Bothell, WA) which was positioned perpendicular to the skin in the medial upper-thigh region. The entire procedure was performed after strict aseptic precautions were taken and skin infiltration (2 ~ 3 mL of 1% lidocaine) was performed with a 100 mm, 17 gauge, insulated nerve block needle and a 19 gauge perineural catheter (SonoPlex Stim cannula; Pajunk, Geisingen, Germany).

**Fig. 1:**
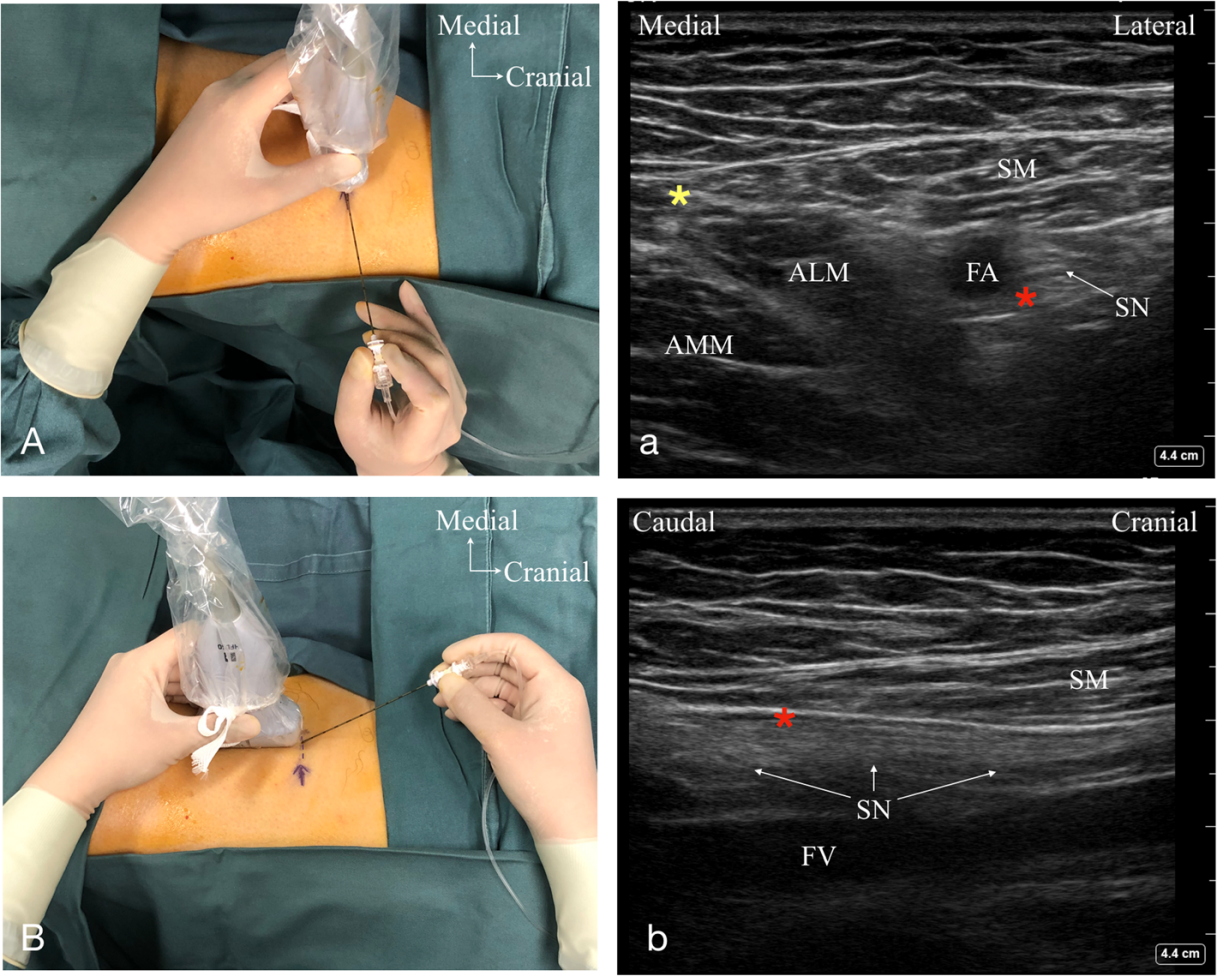
Ultrasound-guided proximal end adductor canal block (ACB) (A/a) and middle ACB (B/b) techniques. (A) Ultrasound probe position of short-axis scanning at the proximal end of the AC and needle orientation for proximal end ACB. (a) Short-axis ultrasound scan image at the proximal end of the AC. (B) Ultrasound probe position of long-axis scanning with the cranial end of the probe aligned with the proximal end of the AC and needle orientation for middle ACB. (b) Long-axis ultrasound scan image with the cranial end of the probe aligned with the proximal end of the AC (at the cranial side in the image). The purple arrow indicates the skin mark of the puncture point for proximal end ACB; the purple dotted line indicates the skin mark of the proximal end of the AC; the red asterisk indicates the endpoint target for the needle tip; the yellow asterisk indicates the alignment of the medial borders of the SM and ALM. ALM, adductor longus muscle; AMM, adductor magnus muscle; FA, femoral artery; FV, femoral venous; SM, sartorius muscle

For subjects randomized to the proximal end group, a short-axis dynamic scan was performed (Fig. 1A). The insertion site was defined by the ultrasound image as the location where the medial margins of the SM and ALM intersected [13] (Fig. 1a). Then, the needle was inserted in-plane in a short-axis lateral-to-medial orientation, through the SM with the final needle tip positioned between the FA and SN (Fig. 1A, a). If the SN could not be well visualized, the needle tip was placed at a 5 o’clock position relative to the FA within the AC [21]. For subjects randomized to the middle group, we used a slightly modified method described by Koscielniak-Nielsen [22]. After identifying the proximal end of the AC in the short-axis view, the ultrasound transducer was rotated 90° to image the SN in the long-axis with the cranial end of the transducer aligned with the proximal end of the AC (Fig. 1B, b). To ensure adequate blinding of the block type to all research personnel performing follow-up evaluations, we choose a needle puncture site at a similar level as in the proximal end group (Fig. 1B). The needle was inserted in-plane in a long-axis with cranial-to-caudal orientation toward the location, 3 ~ 5 cm caudal to the proximal end of the canal, and with the needle tip placed deep into the SM and just superficial to the SN (Fig. 1B, b). If the SN could not be well visualized, the needle tip was placed lateral to the FA within the AC [21].

In both groups, after hydro-dissection with 0.9% saline to confirm proper needle-tip placement within the AC, the perineural catheter was advanced 1 ~ 1.5 cm into the AC under direct ultrasound visualization. After withdrawing the needle, the perineural catheter was tunneled subcutaneously and secured to the upper part of the thigh with surgical glue and an occlusive dressing with an anchoring device. The time between needle skin entry to needle removal was recorded as the block performance time. Ten milliliters of 0.2% ropivacaine was injected as the loading dose via the catheter after negative aspiration. Catheter insertion success was defined as a decrease in the cutaneous sensation to pinprick in the SN distribution area over the ipsilateral medial calf within 30 min after injection. Subjects with a failed catheter insertion or misplaced catheter indicated by a lack of sensory change had their catheter replaced or were withdrawn from the study.

### Intraoperative management

A bispectral index (BIS) monitor was connected for all patients. General anesthesia was induced with intravenous midazolam (1 mg), fentanyl (2 μg/kg), propofol (1.5 ~ 2.0 mg/kg), and rocuronium (0.6 mg/kg). All patients received laryngeal mask airway intubation. Anesthesia was maintained with a sevoflurane and O_2_-N_2_O mixture to keep the BIS within 40 ~ 60. Intravenous fentanyl (1 μg /kg) and rocuronium bromide (0.6–0.9 mg/kg) were administered intraoperatively as needed. On completion of surgery, sevoflurane and N_2_O were discontinued and the neuromuscular blockade was reversed using neostigmine (50 μg/kg) and atropine (20 μg/kg). Extubation was carried out when patients were fully awake.

### Postoperative analgesia

Continuous ACB was initiated immediately after surgery in both groups using an electronic pump (Gemstar, Hospiria Inc., USA) to administer 0.2% ropivacaine at a rate of 6 ml/h through the catheter. PCIA was commenced using a pump set (Gemstar, Hospiria Inc., USA) to deliver boluses of 1.5 ~ 2 μg sufentanil with a 5-min lockout interval and no background infusion. The maximum permitted dosage of sufentanil was set at 8 μg/h. Continuous ACB and PCIA were continued until 48 h after the surgery in both groups. Intravenous parecoxib sodium (40 mg), Q12 h, was administered for 3 days postoperatively.

### Outcomes and data collection

Patients were evaluated postoperatively at 0, 2, 4, 8, 12, 24, and 48 h. The primary outcome measure was the 24 h sufentanil consumption after surgery. The secondary outcome measures included sufentanil consumption at other postoperative time points; pain intensity both at rest and upon passive knee extension to 60° assessed with the NRS score; quadriceps motor strength assessed by a physiotherapist using Lovett’s 6-point scale (0 = no voluntary contraction possible, 1 = muscle flicker, but no movement of limb, 2 = active movement only with gravity eliminated, 3 = movement against gravity but without resistance, 4 = movement possible against some resistance and 5 = normal motor strength against resistance) preoperatively and postoperatively [23]; time to ambulation after surgery defined as the time from the end of surgery until ambulation assisted by a walker or ward nurse; episodes of PONV within 48 h after surgery; patient’s satisfaction with anesthesia and analgesia, which were separately assessed at 48 h using a 5-point scale (5, very satisfied; 4, satisfied; 3, neither satisfied nor dissatisfied; 2, dissatisfied; 1, very dissatisfied); and block-related complications including puncture point infection, leakage, catheter dislodgment, and falling down. The durations of postoperative length of stay were also retrieved from electronic medical records.

### Sample size

The sample size requirement was calculated based on a pilot study (*n* = 10) performed at our institution between January 2019 and February 2019 in which the mean (standard deviation, SD) cumulative 24 h sufentanil consumption after TKA was 0.235 (0.172) μg/kg in the proximal end group and 0.376 (0.188) μg/kg in the middle group. A sample size of 28 patients would be needed for a power (1-beta) of 0.80 and a significance level (alpha) of 0.05. Since it is presumed that 24 h sufentanil consumption may not follow a normal distribution, and since a calculation which assumes a normal distribution might underestimate the sample size, we planned to enroll 31 patients per group.

### Statistical analysis

The statistical analyses were performed using SPSS version 15.0 (SPSS Inc., Chicago, IL, USA). Variables and demographics that followed a normal distribution are expressed as the mean (standard deviation) and were analyzed using a Student’s *t*-test. Variables that did not follow a normal distribution are presented as the median (interquartile range, IQR) and were analyzed using the Mann-Whitney U test. Categorical data are reported as the proportion or percentage and were analyzed using the Chi-squared test. *P*-values < 0.05 (two-sided) were considered statistically significant.

## Results

**Fig. 2:**
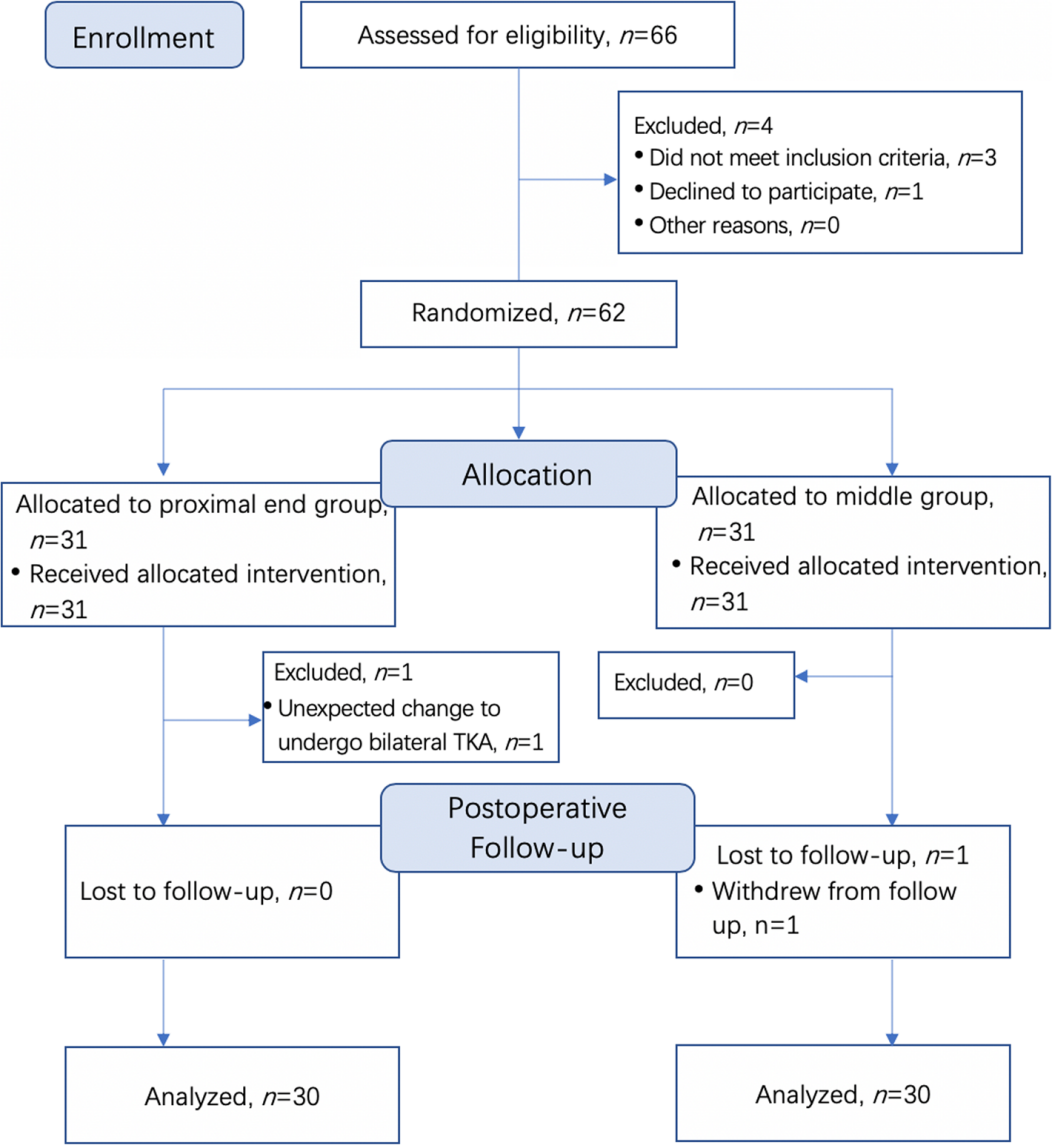
CONSORT patient flowchart

Of the 66 subjects who were approached, 2 (3.03%) did not meet the inclusion criteria (1 patient’s BMI was > 40 kg/m^2^, and 1 patient received tramadol tablets for osteoarthritic knee pain); additionally, 2 (3.03%) patients refused to participate. The remaining 62 subjects were randomly assigned to one of the study groups. One subject who was randomized to the proximal end group unexpectedly needed to undergo bilateral TKA and 1 subject who was randomized to the middle group withdrew from the study during the postoperative follow-up period. Sixty subjects, including 30 in each group with no clinically relevant differences noted between the groups (Table 1) were included in the final analysis (Fig. 2).

**Table 1:** Demographics, preoperative, and intraoperative data

	Proximal end (*n* = 30)	Middle (*n* = 30)
Demographic data		
Age (years), mean (SD)	68.60 (6.20)	67.47 (6.37)
Female Sex, n (%)	26 (86.67%)	25 (83.33%)
BMI (kg/m^2^), mean (SD)	25.27 (3.50)	25.80 (2.28)
ASA-PS class (I/II/III), n	5/24/1	5/25/0
Preoperative data		
NRS score at rest, median (IQR)	0 (0–3)	0 (0–3)
NRS score with activity, median (IQR)	5 (3–6)	5 (4–6)
Quadriceps strength score, median (IQR)	5 (5–5)	5 (5–5)
Time to complete the block and catheter insertion (sec), mean (SD)	144.00 (69.86)	136.37 (84.74)
Intraoperative data		
Operation duration (min), mean (SD)	86.57 (28.71)	93.73 (19.90)
Intraoperative fentanyl (μg/kg), mean (SD)	3.32 (1.21)	3.46 (1.20)

*ASA-PS* American Society of Anesthesiologists-physical status, *SD* Standard deviation, *IQR* Interquartile range

### Primary outcome

The median (IQR) 24 h sufentanil consumption was significantly lower in the proximal end group than in the middle group [0.22 (0.15–0.40) vs. 0.39 (0.23–0.52) μg/kg, *P* = 0.026] (Table 2).

**Table 2:** Cumulative sufentanil consumption (μg/kg) after surgery for both groups

	Proximal end (*n* = 30)	Middle (*n* = 30)	*P* value
Cumulative sufentanil consumption (μg/kg) at different time points			
Primary outcome			
24 h	0.22 (0.15–0.40)	0.39 (0.23–0.52)	*0.026*
Secondary outcomes			
2 h	0 (0–0.04)	0.02 (0–0.07)	*0.222*
4 h	0.03 (0–0.08)	0.07 (0–0.21)	*0.143*
8 h	0.06 (0–0.18)	0.21 (0.10–0.44)	*0.001*
48 h	0.43 (0.23–0.74)	0.59 (0.41–0.89)	*0.031*
Cumulative sufentanil consumption (μg/kg) at different time intervals			
8 h-to-24 h	0.13 (0.07–0.17)	0.10 (0.05–0.19)	*0.525*
8 h-to-48 h	0.38 (0.22–0.50)	0.38 (0.19–0.52)	*0.842*
24 h-to-48 h	0.17 (0.08–0.36)	0.21 (0.11–0.46)	*0.280*

Data are presented as the median (interquartile range)

### Secondary outcomes

Sufentanil consumption was also significantly lower in the proximal end group than in the middle group at 8 h [0.06 (0–0.18) vs. 0.21 (0.10–0.44) μg/kg, *P* = 0.001] and 48 h [0.43(0.23–0.74) vs. 0.59 (0.41–0.89) μg/kg, *P* = 0.031] postoperatively (Table 2). To clarify whether the cumulative sufentanil difference at 24 h and 48 h could be the representation of the initial 8 h difference which is carried forwardly, we also compared the difference of sufentanil consumption during the 8 h -to-24 h, 8 h -to-48 h and 24 h-to 48 h time intervals (Table 3), and the result did not show significant difference between groups (*P*s > 0.05). There were no significant differences in median NRS scores (at rest/upon passive flexion of the operated knee) or quadriceps strength scores assessed at 0, 2, 4, 8, 24, and 48 h postoperatively (*P*s > 0.05) between groups (Table 3, Table 4). The two treatment groups also did not differ significantly in terms of episodes of PONV within 48 h after surgery, time to ambulation, satisfaction scores with anesthesia and analgesia assessed 48 h after surgery, or postoperative length of hospital stay (*P*s > 0.05) (Table 4).

**Table 3:** Postoperative pain NRS scores at each time point for both groups

	Proximal end (*n* = 30)	Middle (*n* = 30)	*P* value
NRS at rest, median (IQR)			
0 h	0 (0–3)	0.5 (0–3)	*0.753*
2 h	0.5 (0–3)	1.5 (0–3)	*0.906*
4 h	0.5 (0–2.63)	1.5 (0–3)	*0.488*
8 h	0 (0–2.0)	1 (0–2.0)	*0.567*
24 h	0.5 (0–3.25)	1 (0–3)	*0.798*
48 h	0 (0–2)	0 (0–1)	*0.165*
NRS upon passive flexion of the operated knee to 60°, median (IQR)			
0 h	2 (0–5)	2.5 (0–4.25)	*0.861*
2 h	3 (0–6)	3 (2–5)	*0.625*
4 h	2 (0–4)	2 (1.75–4.25)	*0.447*
8 h	2 (0–4)	2.5 (0–4)	*0.815*
24 h	3 (0.75–5)	3 (2–5)	*0.788*
48 h	2.5 (1–4)	3 (1–3.25)	*0.845*

*IQR* Interquartile range, *NRS* Numerical rating scale

**Table 4:** Postoperative recovery related data for both groups

	Proximal end (*n* = 30)	Middle (*n* = 30)	*P*
Quadriceps motor strength scores, median (IQR)			
0 h	3 (1–3)	3 (2–3)	*0.513*
2 h	3 (2–4)	3.25 (1.75–4)	*0.477*
4 h	3.5 (2–4)	3.75 (3–4)	*0.486*
8 h	4 (3–4)	4 (3–5)	*0.684*
24 h	4 (3–5)	4.5 (3.88–5)	*0.332*
48 h	4.75 (4–5)	5 (4–5)	*0.356*
Incidence of PONV within 48 h, median (IQR)	0 (0–0)	0 (0–1)	*0.412*
Time to ambulation (h), mean (SD)	39.53 (13.11)	42.01 (17.13)	*0.532*
Satisfaction score with anesthesia assessed at 48 h, median (IQR)	5 (5–5)	5 (4.75–5)	*0.629*
Satisfaction score with analgesia assessed at 48 h, median (IQR)	5 (5–5)	5 (4–5)	*0.412*
Block related complications			
Puncture point infection, n	0	0	–
Leakage, n	1	0	–
Catheter dislodgment, n	0	0	–
Falling down, n	0	0	–
Postoperative LOS (days), mean (SD)	5.46 (2.76)	5.72 (1.94)	*0.680*

*IQR* Interquartile range, *LOS* Length of stay, *PONV* Postoperative nausea and vomiting, *SD* Standard deviation

All continuous ACBs were successful. No infection at the catheter insertion sites or dislodgment of the catheter were reported. Only one case of insertion site leakage was found in the proximal end group. There were also no reported falls secondary to quadriceps weakness.

## Discussion

The main finding of this study was that continuous ACBs performed at the proximal end of the AC in comparison to that at the middle of the AC showed a superior opioid-sparing effect 24 h after TKA; in addition, both ACB locations had a similar influence on the strength of the quadriceps.

To our best knowledge, this is the first clinical RCT compares a continuous ACB performed at the proximal end of the AC (where the medial border of the SM intersects the medial border of the ALM) with a middle AC injection. The underlying mechanism of the current result could be explained by a more recent anatomical study by Tran published after the initiation of the present trial [24]. In his study, following a proximal end AC injection with 10 ml of dye in seven lightly embalmed specimens, they found that the dye spread consistently stained the SN, posteromedial branch of the VMN, superior medial genicular nerve and the genicular branch of the obturator nerve, which are sensory nerves that innervate the knee joint [24]. Instead, cadaveric studies using a distal AC injection failed to report staining of the posteromedial branch of NVM and/or its distal branch, the superomedial genicular nerve [19, 20]. We also found the superior analgesic effect of proximal end AC block could only be obviously observed till 8 h after surgery. We suppose this could be due to the effect of the initial loading dose of ropivacaine. A 10 ml injection of 0.2% ropivacaine at the middle of the AC may spread cephalad toward the proximal end of the AC and as a result provide similar analgesia at least during the first 4 h after surgery. Following that, when the analgesic effect of the initial dose wore off, ‘rebound pain’ may have occurred and induced ‘rebound’ opioid consumption requirements [25, 26], as shown at the 8 h time point in the middle ACB group in this study. The initial 8 h difference might have also carried forwardly till 48 h after surgery in the current study, since the difference of opioid consumption during the 8 h -to-24 h, 8 h-to-48 h and 24 h-to 48 h time interval did not show significance. This phenomenon indicates that a high volume of single injection at the middle AC may produce similar analgesia at the early period immediately after TKA, while a continuous low volume infusion at the proximal end of AC could provide consistent and prolonged pain relieve during the following period.

In studies aiming to clarify the optimal location to maintain ACB after TKA, three previously published RCTs by Mariano [5], Romano [6] and Meier [7] had investigated the “proximal AC” and “distal AC” and failed to detect significant differences in regard to 24 h postoperative opioid consumption, as well as in quadriceps strength or motor function. The discrepancies between the present study and these three RCTs can likely be attributed to the different definitions of the AC [5–7]. Base on their description, these studies actually compared the distal FT [5, 6] or the proximal AC [7] with a more cephalad injection in the FT [5–7], instead of the distal AC with the proximal AC. In another study with the similar purposes, Sztain8 compared the analgesic effect of continuous ACB at the mid-thigh level (termed “proximal AC” in their study), defined as the midpoint between the anterior superior iliac spine and the patella [12, 27, 28] which recently has been proved to actually indicate a cranial location to the proximal end of AC and inside the distal FT in most subjects [11], with a more distal insertion closer to the adductor hiatus. The result showed the mid-thigh level block provide improved analgesic effect after TKA. Both the study by Sztain [8] and the current study provided clinical evidence supporting previous speculation that, instead of a true AC, a distal TF or a proximal end AC block would be more suitable to alleviate pain after knee surgery [10, 13, 20].

The ideal location for continuous ACB after TKA is supposed to be where it achieves maximum analgesia with minimal quadriceps weakness. The current study did not show a significant difference in the effect of catheter locations on quadriceps strength measured manually by a physiotherapist on a Lovett’s scale. This could also be explained by the finding of the latest cadaveric study by Tran [24], where the proximal end AC injection (10 ml, which is the same volume as the loading dose in the present study) was found to spare the anterior branches of the NVM which would likely preserve greater vastus medialis activation, contributing to the quadriceps motor sparing characteristic of the proximal ACB. Another non-negligible contributor could be the following blockade infusion (at a rate of 6 ml/h) regimen adopted in the current study which may avoid further cephalad spread of the local anesthetic following the initial dose to the motor component of the femoral nerve [29]. A further study powered to explore the effect of catheter location on quadriceps motor function is needed.

The current study had some limitations. First, the quadriceps muscle strength was only evaluated manually by a physiotherapist on a Lovett’s scale, which is not as precise as by using the force dynamometer such as the measurement of maximum voluntary isometric contraction [7, 29]. In addition, we did not implement a validated test to measure patient mobilization ability, such as the Timed “Up and Go” measurement [30], which could directly reflect the balance between “pain-control during movement” and “preserving strength” that is important for effective pain management after TKA [31]. The current study is unable to show whether continuous infusion will increase blockade related side effects. Comparing the analgesic effect and safety of the single shot ACB, continuous ACB without single shot initiation, and single shot initiation followed by continuous infusion is not the primary interests of the present work, but clearly warrants further studies. Finally, as this is a single-center study with a small sample size which is limited to TKA patients, the results may not be generalizable to other types of knee procedures.

## Conclusions

In conclusion, this study demonstrates that continuous ACB at the proximal end of the AC—the location on ultrasound where the medial margins of the SM and ALM intersect—provides a better analgesic effect without significantly compromising quadriceps motor strength compared to that at the middle of the AC after TKA. These results confirm the findings reported by the latest cadaveric study on the neuroanatomy of the AC. Moreover, it also indicates that a true ACB may not produce the effective analgesia, instead, a proximal end AC might be a more suitable block to alleviate pain after TKA, which enables informed choices for further RCTs.

## Data Availability

The data supporting the conclusions of this article are available at: 10.17632/hvgg35pz5k.1

## Abbreviations

AC: Abbductor canal
ACB: Adductor canal block
ALM: Adductor longors muscle
ASA: American Society of Anesthesiologists
BIS: Bispectral index
BMI: Body mass index
FA: Femoral artery
FT: Femoral triangle
IQR: Interquartile range
NRS: Numerical rating scale
NVM: Nerve to vastus medialis
PCIA: Patient-controlled intravenous analgesia
RCT: Randomized clinical trial
SD: Standard deviation
SM: Sartorius muscle
SN: Saphenous nerve
TKA: Total knee anthroplasty
